# Quack Medicines

**Published:** 1898-02-12

**Authors:** 


### The Hospital

A Journal of

The Medical Sciences and Hospital Administration.

Vol. XXIII.—No. 594]. Edited by Sir Henry Burdett, K.C.B., and Solomon C. Smith, M.D., M.R.C.P. [Feb. 12, 1898.

### Quack Medicines.

The law of England, which is quite sufficiently severe upon many common methods of obtaining money by false pretences, is not only strangely indifferent with regard to others, but even appears to fall short of fulfilling the reasonable demands of ignorant people to be protected against certain forms of fraud. A man who sells to some simpleton a worthless ring by means of a declaration that it is precious, is properly treated as a rogue and a vagabond; but a man who puts a little of any cheap and common drug into a bottle of flavoured water, announces that it will cure all diseases, and offers it for sale at a thousand times its value, is not only permitted to escape scot free, but is even taken under the special protection of the Chancellor of the Exchequer, and is granted a monopoly of sale for his rubbish in exchange for a contribution to the national revenue. We have heard it stated, on the basis of the amount of duty paid for quack medicines, that they are purchased in the Queen's dominions to the extent of three million pounds sterling annually; and the statement is at least an approximation to the truth. The reputation of any one of these preparations is usually ephemeral; and hence it has become a common practice for the "proprietor," as soon as a certain popularity has been obtained by advertising, to dispose of his "property" to a joint stock company, taking payment in anything except shares, and relieving himself as speedily as possible of all responsibility for his compound. The unfortunate shareholders soon find that the demand for their commodity has passed away, but are seldom on this account less disposed to invest in the next nostrum which may come into the market. It is strange how deeply the power of believing in quacks and in their preparations is rooted in the English race. Its manifestations date from an early period of our history; and, in comparatively recent times, have furnished an endless theme for satire. Goldsmith, for example, in his "Citizen of the World," declares that there must be something strangely obstinate in an English patient, who refuses the health which is everywhere offered to him on such easy terms. "Does he take a pride in being bloated with a dropsy? does he find pleasure in the alternations of an intermittent fever? or feel as much satisfaction in nursing up his gout, as he found pleasure in acquiring it?" Of the inventors of the innumerable specifics, he tells us that many "have spent a great part of their lives unconscious of any latent excellence (in the art of healing) till a bankruptcy, or a residence in jail, have called their miraculous powers into exertion." And so the game continues; resting upon the notorious fact that the British public will swallow anything, or will rub themselves with anything, the efficacy of which is set forth in announcements sufficiently conspicuous and continued, but resting upon no known authority and supported by no tittle of evidence. Many of the most widely advertised quack medicines are prepared from prescriptions which have been given by physicians for actual cases, and are capable of doing as much mischief to persons for whom they are unsuitable as good under opposite conditions. One of them, for example, contains potassium iodide as its only active ingredient, and may often be useful in certain forms of constitutional syphilis. It is, nevertheless, manifest that, even for these, the iodide would be far more useful if it were prescribed with reference to personal conditions; while in non-syphilitic cases it may work much mischief upon some of those who swallow it.

They "manage these matters better in France," where the sale of proprietary remedies is only permitted under strict supervision from the Academy of Medicine, and where the public is therefore much better protected than with us. There was an amusing lawsuit a few years ago, in which the late Mr. Holloway, of pill and ointment notoriety, was the defendant. He had been expressing his great desire to obtain a license for the sale of his "remedies" in France, and the plaintiff in the action undertook to procure this license for a specified fee. He complied with certain formalities, and obtained a license for the sale of *La pommade dite Holloway*, described as consisting of so much beeswax, so much resin, and so much suet, or similar harmless materials. A license which disclosed the simple composition of the stuff was useless to the vendor, and Mr. Holloway refused to pay the promised fee until a judgment against him had been obtained. We trust, whenever matters medical come again under the consideration of Parliament, that our own public analysts will be empowered to deal with advertised preparations in a somewhat similar manner, and to relieve the country from the disgrace of accepting, as any portion of its revenue, a contribution from the profits of unscrupulous vendors.

